# Accuracy and precision of non-invasive cardiac output monitoring by electrical cardiometry: a systematic review and meta-analysis

**DOI:** 10.1007/s10877-019-00330-y

**Published:** 2019-06-07

**Authors:** M. Sanders, S. Servaas, C. Slagt

**Affiliations:** Department of Anesthesiology, Pain and Palliative Medicine, Radboud University Medical Center, Geert Grooteplein Zuid 10, 6500 HB Nijmegen, The Netherlands

**Keywords:** Hemodynamic monitoring, Cardiac output, Electrical cardiometry, Electrical velocimetry, Bioimpedance, Non-invasive, Systematic review, Meta-analysis

## Abstract

Cardiac output monitoring is used in critically ill and high-risk surgical patients. Intermittent pulmonary artery thermodilution and transpulmonary thermodilution, considered the gold standard, are invasive and linked to complications. Therefore, many non-invasive cardiac output devices have been developed and studied. One of those is electrical cardiometry. The results of validation studies are conflicting, which emphasize the need for definitive validation of accuracy and precision. We performed a database search of PubMed, Embase, Web of Science and the Cochrane Library of Clinical Trials to identify studies comparing cardiac output measurement by electrical cardiometry and a reference method. Pooled bias, limits of agreement (LoA) and mean percentage error (MPE) were calculated using a random-effects model. A pooled MPE of less than 30% was considered clinically acceptable. A total of 13 studies in adults (620 patients) and 11 studies in pediatrics (603 patients) were included. For adults, pooled bias was 0.03 L min^−1^ [95% CI − 0.23; 0.29], LoA − 2.78 to 2.84 L min^−1^ and MPE 48.0%. For pediatrics, pooled bias was − 0.02 L min^−1^ [95% CI − 0.09; 0.05], LoA − 1.22 to 1.18 L min^−1^ and MPE 42.0%. Inter-study heterogeneity was high for both adults (I^2^ = 93%, p < 0.0001) and pediatrics (I^2^ = 86%, p < 0.0001). Despite the low bias for both adults and pediatrics, the MPE was not clinically acceptable. Electrical cardiometry cannot replace thermodilution and transthoracic echocardiography for the measurement of absolute cardiac output values. Future research should explore it’s clinical use and indications.

## Introduction

### Rationale

Information about the hemodynamic status of patients plays an important role in daily clinical practise in the emergency department, the intensive care unit (ICU) and operating room (OR). Heart rate, blood pressure and pulse-oximetry monitoring is generally applied. Advanced hemodynamic monitoring is used in critically ill and high-risk surgical patients. Many studies, including meta-analyses [1–5], have shown that optimization of hemodynamic parameters reduces mortality, morbidity, post-operative complication rates, duration of hospital stay and improves functional recovery in high-risk surgical patients.

In adults intermittent pulmonary artery thermodilution (intermittent PAC) and transpulmonary thermodilution (TPTD) are considered the gold standard for the measurement of cardiac output (CO). However, these methods are invasive and linked to complications [6–9]. In neonates and pediatric patients transthoracic echocardiography (TTE) is the most commonly used technique. This technique has several limitations as it requires an experienced operator, is technically demanding and is obtained intermittently. Recently, many non-invasive devices have been developed and studied [10–12].

One of these new non-invasive, yet to become established, methods is thoracic electrical bioimpedance (TEB), first described in 1966 by Kubicek and colleagues [13]. This method is based on changes in thoracic resistance as a result of changes in blood velocity during the cardiac cycle and uses an algorithm to calculate the CO. Sramek and Bernstein (1986) modified the algorithm [14]. The most recent modification is the Bernstein-Osypka Eq. (2003), also called electrical velocimetry or electrical cardiometry (EC) [15, 16]. The latter name will be used in this manuscript.

**Fig. 1:**
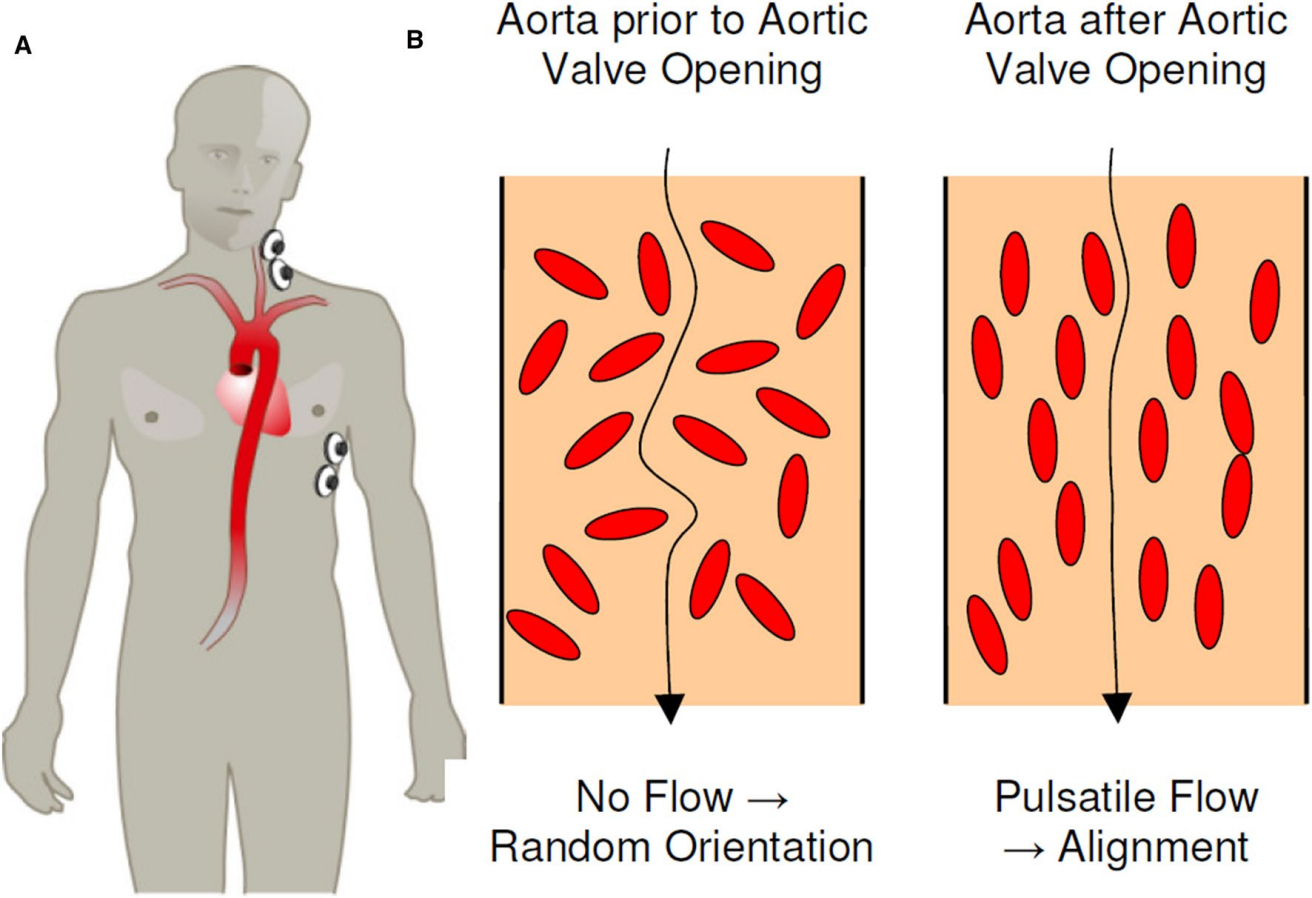
**a** Placement of electrodes on the left base of the neck and on the left inferior side of the thorax at the level of the xiphoid process. **b** Arrangement and orientation of erythrocytes during diastole (left) and systole (right) explaining the difference in thoracic impedance. Figure reproduced from Osypka Medical GmbH, an introduction to Electrical Cardiometry [19]

EC measures alteration in thoracic resistance or impedance, using four skin electrodes. EC is able to isolate the changes in impedance created by the circulation, partly caused by the change in orientation of the erythrocytes during the cardiac cycle (Fig. 1). Impedance cardiography can be affected by the remaining thoracic tissue or fluid [17]. Two electrodes are placed on the left base of the neck and two on the left inferior side of the thorax at the level of the xiphoid process (Fig. 1). Exact placement of the electrodes is important because measurements can vary when placement is incorrect. The inter-electrode gap of the lower electrodes should be 15 cm in adults [18]. The electrodes are connected to either the Aesculon^®^ monitor (Osypka Medical GmbH, Berlin, Germany) or the ICON^®^ monitor (Osypka Medical GmbH, Berlin, Germany), which is smaller in size and portable. Both devices derive stroke volume, heart rate and CO from the impedance values. Further details of the devices are described elsewhere [15, 16, 19].

This safe and easy applicable method could be a suitable candidate to complement or replace invasive CO monitoring. Several studies tried to validate EC using different reference methods, leading to conflicting results. EC was part of three meta-analyses with limited studies only [10–12]. So, its place between all existing hemodynamic monitoring devices has yet to be determined. Our meta-analysis focuses exclusively on EC, for definitive validation of accuracy and precision in both adults and pediatrics.

### Objective

We conducted a systematic review to assess the accuracy and precision of CO measurement by EC compared to a reference method, in both adults and pediatrics. The primary outcome measures were (i) accuracy, defined as the bias between the CO measured by EC and the reference methods, (ii) precision, defined as the standard deviation (SD) of the bias, (iii) the limits of agreement (LoA) defined as [bias ± 1.96*SD], and (iv) the mean percentage error (MPE) derived from the SD and mean CO. A pooled MPE of less than 30% was considered clinically acceptable, as described by Critchley and Critchley [20].

## Methods

This systematic review was conducted using Preferred Reporting Items for Systematic Reviews and Meta-analyses (PRISMA) approach (See Table 5 in Appendix 1) [21].

### Eligibility criteria

Eligibility criteria were (1) studies comparing CO measurement by EC and a reference method, (2) studies using Bland–Altman analysis to report bias, SD of the bias and MPE or for which those data could be extracted [22], (3) studies performed in humans and (4) studies published as a full paper in English. Studies involving participants of any age and under any clinical circumstances were included. No restriction in publication date was applied.

### Information sources and search

Two independent investigators (MS and SS) performed an electronical database search of PubMed, Embase, Web of Science and the Cochrane Library of Clinical Trials. The last date of search was January 4, 2019. Studies that were not published as full journal articles (e.g. letters, editorials, conference papers) and retracted publications were excluded. The search strategy conducted in PubMed is shown in Appendix 2. The search strategies for the other databases were comparable and are available on request. The manufacturer of ICON^®^/Aesculon^®^ (Osypka Medical GmbH, Berlin, Germany) and the website were consulted to identify additional studies. The reference lists of all included studies were screened for additional studies. EndNote^®^ software, version X8.1 (Thomson Reuters, New York, USA) was used to arrange all articles and to filter the duplicates between databases.

### Study selection

**Fig. 2:**
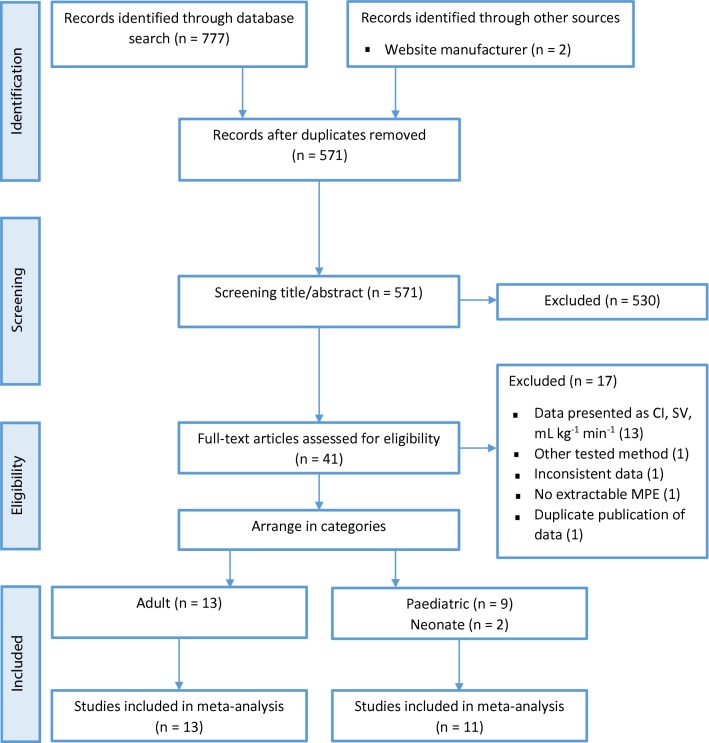
Flow diagram of the study selection process. *CI* cardiac index, *MPE* mean percentage error, *SV* stroke volume

Two independent investigators (MS and SS) identified the potentially relevant studies. The first selection was based on title and abstract. The remaining full text articles were reviewed for eligibility. After including an article we arranged them in the category adult or pediatric patients. Conflicts were resolved by consensus or after consultation with the third investigator (CS). The flow diagram of this study selection process is shown in Fig. 2.

### Data collection process

A customized data form was developed by three investigators (MS, SS and CS), using Microsoft Excel (Microsoft Office, Washington, USA). The data extraction form was pilot-tested on five randomly-selected included studies and refined. Data were extracted independently by two investigators (MS and SS). Patient characteristics, clinical setting, age, reference method and device, number of patients, total number of measurements, and financial support were considered relevant (Tables 1, 2). For the statistical analyses we extracted mean CO, CO range, bias, SD of the bias, LoA and MPE (See Tables 6, 7 in Appendix 3, 4). Precision of the reference and tested method and assessment of trending ability were added to the data extraction form after the pilot-test. Disagreements in data extraction were resolved by consensus or by consultation of CS.

**Table 1:** Study characteristics of included adult studies

Authors	Year	Patient characteristics	Clinical setting	Age, mean ± SD (range)	ICON/Aesculon	Reference method	Sample size	Financial support
Heringlake [30]	2007	Cardiac surgery	OR and ICU	66 ± 11	Aesculon	PACi	29	Osypka Medical and Edwards Lifesciences
Magliocca [31]	2017	Liver transplantation	OR	58 ± 8	ICON	PACi	19	ICON provided by the manufacturer
Malik [29]	2014	Cardiac surgery	OR	54.6 (41–67)	ICON	PACc	60	Research grant
Martin [32]	2016	Pregnant women (≥ 24 weeks GA)	Outpatient unit	31 ± 6.0, )19–42)	ICON	TTE	44	Research grant
Mekis [33]	2008	Cardiac surgery	OR and ICU	68.5 ± 7.6 (49–78)	Aesculon	PAC	16	na
Petter [34]	2011	Diagnostic right heart catheterization	Cardiology unit	59 ± 2.7	Aesculon	PACi	33	Research grant
Rajput [28]	2014	Cardiac surgery	OR	62.32 ± 5.12	ICON	PACi	25	na
Raue [35]	2009	SIRS or sepsis post-surgery	ICU	63 (23–93)	Aesculon	TPTD	30	Aesculon provided by the manufacturer
Schmidt [36]	2005	Cardiac surgery	OR	65.8 (43–81)	Aesculon	TEE	37	na
Trinkmann [38]	2011	Hemodynamically stable cardiac patients	Cardiology unit	54 ± 17 (15–86)	Aesculon	Fick	120	None
Trinkmann [37]	2016	Hemodynamically stable cardiac patients	Cardiology unit	Median 53 (15–83)	Aesculon	MRI	134	None
Wang [39]	2018	Liver transplantation	OR	56 ± 7 (41–68)	Aesculon	PACc	23	Research grant
Zoremba [40]	2007	Critically ill post-surgery	ICU	64.6 (34–83)	Aesculon	PACi and TPTD	50	na

*GA* gestational age, *ICU* intensive care unit, *MRI* magnetic resonance imaging, *na* not available, *OR* operating room, *PACc* continuous pulmonary artery thermodilution, *PACi* intermittent pulmonary artery thermodilution, *SD* standard deviation, *SIRS* systemic inflammatory response syndrome, *TEE* transesophageal echocardiography, *TPTD* transpulmonary thermodilution, *TTE* transthoracic echocardiography

**Table 2:** Study characteristics of included pediatric studies

Authors	Year	Patient characteristics	Clinical setting	Age, mean ± SD (range)	ICON/Aesculon	Reference method	Sample size	Financial support
Altamirano-Diaz [42]	2017	Obese and normal weight pediatric patients	Outpatient unit	Median 12.3 (5.1–17.8)	ICON	TTE	131	Research grant
Altamirano-Diaz [41]	2018	Post cardiac surgery	Outpatient unit	12.3	ICON	TTE	49	Research grants
Chaiyakulsil [43]	2018	Critically ill	ICU	4.9 ± 4.6	ICON	TTE	121	na
Kusumastuti [44]	2015	Cardiac surgery	ICU	Median 39.50 weeks	Aesculon	TTE	30	None
Lotfy [45]	2018	Biliary atresia, kasai procedure	OR	73 (58–86) days	ICON	TEE	42	None
Noori [46]	2012	Healthy term neonates	NICU	GA 39.2 ± 1.1 weeks	Aesculon	TTE	20	None
Norozi [47]	2008	Cardiac catheterization	OR	3.4 (12 days–17.8 year)	Aesculon	Fick	32	na
Rauch [48]	2013	Obese pediatric patients	Outpatient unit	Median 12.52 (7.9–17.6)	ICON	TTE	64	Research grants
Tomaske [50]	2008	Cardiac catheterization	OR	7.8 (0.5–16.5)	Aesculon	PACi	50	Research grants
Tomaske [49]	2009	Cardiac catheterization	OR	Median 5.7 (0.5–16)	Aesculon	TTE	36	Research grants
Torigoe [51]	2015	Low birth weight neonates	NICU	GA 32 (25–37) weeks	Aesculon	TTE	20	None

*GA* gestational age, *na* not available, *NICU* neonatal intensive care unit, *OR* operating room, *PACi* intermittent pulmonary artery thermodilution, *SD* standard deviation, *TEE* transesophageal echocardiography, *TTE* transthoracic echocardiography

Mean CO, bias, LoA, SD, MPE and precision of the reference or tested method were defined according to the following equations:

1 $$Mean\,CO = \frac{Mean\,COec + Mean\,COreference}{2}$$

2 $$Bias = Mean\,COreference - Mean\,COec$$

3 $$Limits\,of\,Agreement = bias \pm 1.96*SD\, {\text{or}}\,SD = \frac{upper\,LoA - lower\,LoA}{1.96*2}$$

4 $$Mean\,Percentage\,Error = \frac{1.96*SD\,of\,bias\,between\,methods}{Mean\,CO}*100\%$$

5 $$Precision\,method x = \frac{1.96*SD\,of\,reproducibility}{mean\,CO\,method\,x}$$

6 $$Precision\,method\,x = 1.96 * Coefficient\,of\,Variation.$$

Missing information was calculated using the equations above. If the data could not be calculated, data was extracted from the Bland–Altman plot. If both options could not be applied, the authors were contacted. Duplicate publication of data was assessed by juxtaposing author names, reference methods, sample sizes, outcome measures mean CO, bias, MPE and data points in Bland–Altman plots.

### Risk of bias assessment in individual studies

To assess the risk of bias for individual studies we used the Quality Assessment of Diagnostic Accuracy Studies (QUADAS-2) guidelines [23]. The original QUADAS-2 tool consists of the four domains patient selection, index test, reference test, flow and timing. Signalling questions are used to assess the risk of bias in each domain. The first three domains are also assessed in terms of concerns about applicability. Kim et al. modified these guidelines to make them more suitable for method-comparison studies [24]. We modified Kim’s QUADAS-2 tool and pilot-tested it on five randomly-selected included studies and refined it accordingly. After the pilot-test, we developed a fifth domain, to assess the statistical analysis and implemented the recommendations of Cecconi [25]. The modified QUADAS-2 tool is available in Table 8 in Appendix 5. MS and SS independently assessed the risk of bias. Conflicts were resolved by consensus or by consultation of CS.

### Summary measures

The primary outcome measures were (i) accuracy, defined as the bias between the CO measured by EC and the reference methods, (ii) precision, defined as the SD of the bias, (iii) the LoA and (iv) the MPE. A pooled MPE of less than 30% was considered clinically acceptable, as described by Critchley and Critchley [20].

### Synthesis of results

Pooled bias, LoA and MPE for both adults and pediatrics were calculated using a random-effects model, as heterogeneity could be present, and forest plots were created. The weight given to the results of the independent studies was determined according to the inverse variance method. Inter-study heterogeneity was calculated using a Q test and described as an I^2^ index (0% no heterogeneity, 25% low heterogeneity, 50% moderate heterogeneity, 75% high heterogeneity) [26]. If an individual study led to multiple outcome measures for bias, LoA and MPE, the outcomes of those studies were presented in different rows in the forest plot.

### Subgroup analyses

Subgroup analyses of the gold standard thermodilution (TD) in adults and most commonly used method TTE in pediatrics were pre-specified for definitive validation of EC. For adults, we distinguished between intermittent TD and continuous TD, as continuous TD averages CO over a longer time period. This led to the subgroups intermittent TD, continuous TD and other reference method. For pediatrics we distinguished between children and neonates, which led to the subgroups TTE children, TTE neonates and other reference method children. A test for subgroup differences was applied. Subgroup analysis for clinical setting was conducted post hoc in adults. This led to the subgroups cardiac surgery, OR, ICU and other clinical setting.

### Risk of publication bias across studies

Risk of publication bias across studies was assessed for both adults and pediatrics using funnel plots, showing the bias versus it’s standard error. The symmetry of the funnel plots was assessed visually and by Egger’s regression test using a significance level of 0.1 [27].

The statistical analyses were conducted using R, version 3.4.2 (R Foundation for Statistical Computing, Vienna, Austria), Rstudio (RStudio, Inc., Boston, USA) and SPSS Statistics, version 25.0 (IBM Business Analytics, New York, USA). The lay-out of the forest and funnel plots was customized using Adobe Photoshop CS4 (Adobe Systems, California, USA).

## Results

### Study selection

We found an initial amount of 777 citations through the database search and two additional records by consultation of the manufacturer’s website [28, 29]. After duplicates were removed, 571 studies remained. After title and abstract screening, 41 studies remained. Those full-text articles were assessed for eligibility, which led to 24 included studies [28–51] and 17 excluded studies [18, 52–67]. The included studies were divided into 13 studies in adults [28–40] and 11 studies in pediatrics [41–51]. Contacting the manufacturer and screening of the reference lists of all included studies led to no additional studies. The flow diagram of the study selection process is shown in Fig. 2. The articles which were excluded after full-text analysis and the reason for exclusion are listed in Appendix 6.

### Study characteristics

Study characteristics of the included studies are presented in Tables 1 and 2. A total of 620 adults and 603 pediatric patients were included. Sample size ranged from 16 to 134 patients with a mean of 52 patients. Concerning adult studies; two were conducted in the OR during liver transplantation surgery [31, 39], three during cardiac surgery [28, 29, 36], two both during cardiac surgery and post cardiac surgery in the ICU [30, 33], two in the ICU [35, 40], three in the cardiology unit [34, 37, 38] and one in the outpatient unit [32]. Concerning pediatric studies; four were conducted in the OR [45, 47, 49, 50], two in the ICU [43, 44], two in the neonatal intensive care unit (NICU) [46, 51], and three in the outpatient unit [41, 42, 48]. The ICON^®^ device was used in nine studies [28, 29, 31, 32, 41–43, 45, 48] and the Aesculon^®^ in fifteen studies [30, 33–40, 44, 46, 47, 49–51]. In the majority of the adult studies intermittent PAC was used as reference method [28, 30, 31, 33, 34, 40]. Other used reference methods in adults were continuous PAC [29, 39], TPTD [35, 40], TTE [32], transesophageal echocardiography (TEE) [36], magnetic resonance imaging (MRI) [37] and Fick-method [38]. The mean age in adults was 51 years. In the pediatric studies the most commonly used reference method was TTE [41–44, 46, 48, 49, 51] except for three studies, which used intermittent PAC [50], TEE [45] and Fick-method [47]. Two studies focussed on neonates with a mean gestational age of 36 weeks, both using TTE as reference method [46, 51]. Three studies acknowledged financial or material support by Osypka Medical GmbH [30, 31, 35].

### Contacting authors

We contacted three authors concerning the direction of the bias (reference–tested method or tested–reference method) [28, 40, 47]. One of them responded [47] and for the other two studies we interpreted the direction of the bias ourselves [28, 40]. We contacted one author concerning the mean CO and MPE [67]. As the mean CO was not described in the manuscript and could not be extracted from the Bland–Altman plot, the MPE could not be calculated. The author did not respond and therefore the study was excluded.

### Risk of bias in individual studies

The assessment of the risk of bias for adult studies is provided in Table 3 and for pediatrics in Table 4. The majority of the included studies was judged low risk of bias with respect to patient selection, tested method, reference method and flow timing. For six studies potential for bias existed in more than one of those four domains, but were considered low risk [30, 33, 34, 37, 38, 47]. Concerning the statistical analysis domain, all studies were judged high risk, except for two studies [46, 49]. Concerns on applicability were assessed low for all studies, which is not shown in Tables 3 and 4.

**Table 3:** Risk of bias for included adult studies, according to the modified QUADAS-2 tool, partly reproduced from Kim et al. [24]

Author	Patient selection		Tested method	Reference method					Flow and timing			Statistical analysis						
	Were subject population of interest and demographic data described?	Were inclusion and exclusion criteria clearly described?	Was the tested method described clearly? (calibration, position and characteristics of monitoring device)	Was the reference method described clearly? (calibration, position and characteristics of monitoring device)	Is the reference method likely to correctly measure cardiac output?	In case of TPTD or intermittent PAC: Was an average of three readings taken for analysis?	In case of echocardiography: Was the reference method assessed by the same, experienced investigator in each patient?	Were the reference method results interpreted without knowledge of the results of the tested method?	Were number of patients enrolled and who dropped out clearly described in the result?	Were the tested method and reference method measured simultaneously?	Was the method of acquiring paired measurement well described?	Do the bias described in the manuscript and in the figures match? (that is, both are [tested method minus reference method] or [reference method minus tested method])	Do the SD described in the manuscript and the LoA in the figures match?	Does the mean percentage error described in the manuscript and recalculated by the reviewers match?	In case of multiple observations per individual, did they apply statistical analysis for repeated measurements?	Was the precision of the reference method measured within the study?		Was the precision of the tested method measured within the study?
Heringlake [30]	+	−	+	+	+	+	na	?	−	−	+	+	+	?	na	−	−	
Magliocca [31]	+	+	+	+	+	+	na	?	+	+	+	+	+	?	+	−	−	
Malik [29]	+	+	+	+	+	na	na	+	+	+	+	−	−	−	+	−	−	
Martin [32]	+	+	+	+	−	na	+	+	+	+	+	+	+	+	−	−	−	
Mekis [33]	+	−	+	+	+	+	na	?	+	+	+	+	+	?	−	−	−	
Petter [34]	−	+	+	+	+	+	na	?	−	+	−	+	+	?	−	−	−	
Rajput [28]	+	+	+	+	+	+	na	?	+	+	+	−	−	+	−	+	+	
Raue [35]	+	+	+	+	+	+	na	?	+	+	+	+	+	?	na	−	−	
Schmidt [36]	+	+	+	+	−	na	+	+	+	+	+	+	+	+	na	−	−	
Trinkmann [38]	+	−	+	+	−	na	na	?	+	−	−	+	+	?	−	+	+	
Trinkmann [37]	+	+	+	+	−	na	na	+	+	−	+	+	+	−	−	−	+	
Wang [39]	+	+	+	+	+	na	na	?	+	+	+	+	+	+	−	−	−	
Zoremba [40]	+	+	+	+	+	+	na	?	+	+	+	−	+	?	na	+	+	

+ yes, − no, ? unclear, *na* not applicable

**Table 4:** Risk of bias for included pediatric studies, according to the modified QUADAS-2 tool, partly reproduced from Kim et al. [24]

Author	Patient selection		Tested method	Reference method					Flow and timing			Statistical analysis					
	Were subject population of interest and demographic data described?	Were inclusion and exclusion criteria clearly described?	Was the tested method described clearly? (calibration, position and characteristics of monitoring device)	Was the reference method described clearly? (calibration, position and characteristics of monitoring device)	Is the reference method likely to correctly measure cardiac output?	In case of TPTD or intermittent PAC: Was an average of three readings taken for analysis?	In case of echocardiography: Was the reference method assessed by the same, experienced investigator in each patient?	Were the reference method results interpreted without knowledge of the results of the tested method?	Were number of patients enrolled and who dropped out clearly described in the result?	Were the tested method and reference method measured simultaneously?	Was the method of acquiring paired measurement well described?	Do the bias described in the manuscript and in the figures match? (that is, both are [tested method minus reference method] or [reference method minus tested method])	Do the SD described in the manuscript and the LoA in the figures match?	Does the mean percentage error described in the manuscript and recalculated by the reviewers match?	In case of multiple observations per individual, did they apply statistical analysis for repeated measurements?	Was the precision of the reference method measured within the study?	Was the precision of the tested method measured within the study?
Altamirano-Diaz [42]	+	+	+	+	+	na	+	+	+	+	+	+	+	+	na	−	−
Altamirano-Diaz [41]	+	+	+	+	+	na	+	?	+	+	+	+	+	?	na	−	−
Chaiyakulsil [43]	+	+	+	+	+	na	−	+	+	+	+	+	−	−	−	−	−
Kusumastuti [44]	+	+	+	+	+	na	−	?	+	+	+	−	+	−	na	−	−
Lotfy [45]	+	+	+	+	−	na	?	?	+	+	+	+	+	?	−	−	−
Noori [46]	+	+	+	+	+	na	+	+	+	+	+	+	+	+	+	−	+
Norozi [47]	+	−	+	+	−	na	na	?	+	+	+	−	+	?	na	−	−
Rauch [48]	+	+	+	+	+	na	+	+	+	+	+	+	+	?	na	−	−
Tomaske [50]	+	+	+	+	+	+	na	+	+	+	+	+	+	−	−	−	−
Tomaske [49]	+	+	+	+	+	na	+	+	+	+	+	+	+	?	na	+	+
Torigoe [51]	+	+	+	+	+	na	+	+	+	+	−	+	+	+	−	−	−

+ yes, − no, ? unclear, *na* not applicable

### Synthesis of results, adults

**Fig. 3:**
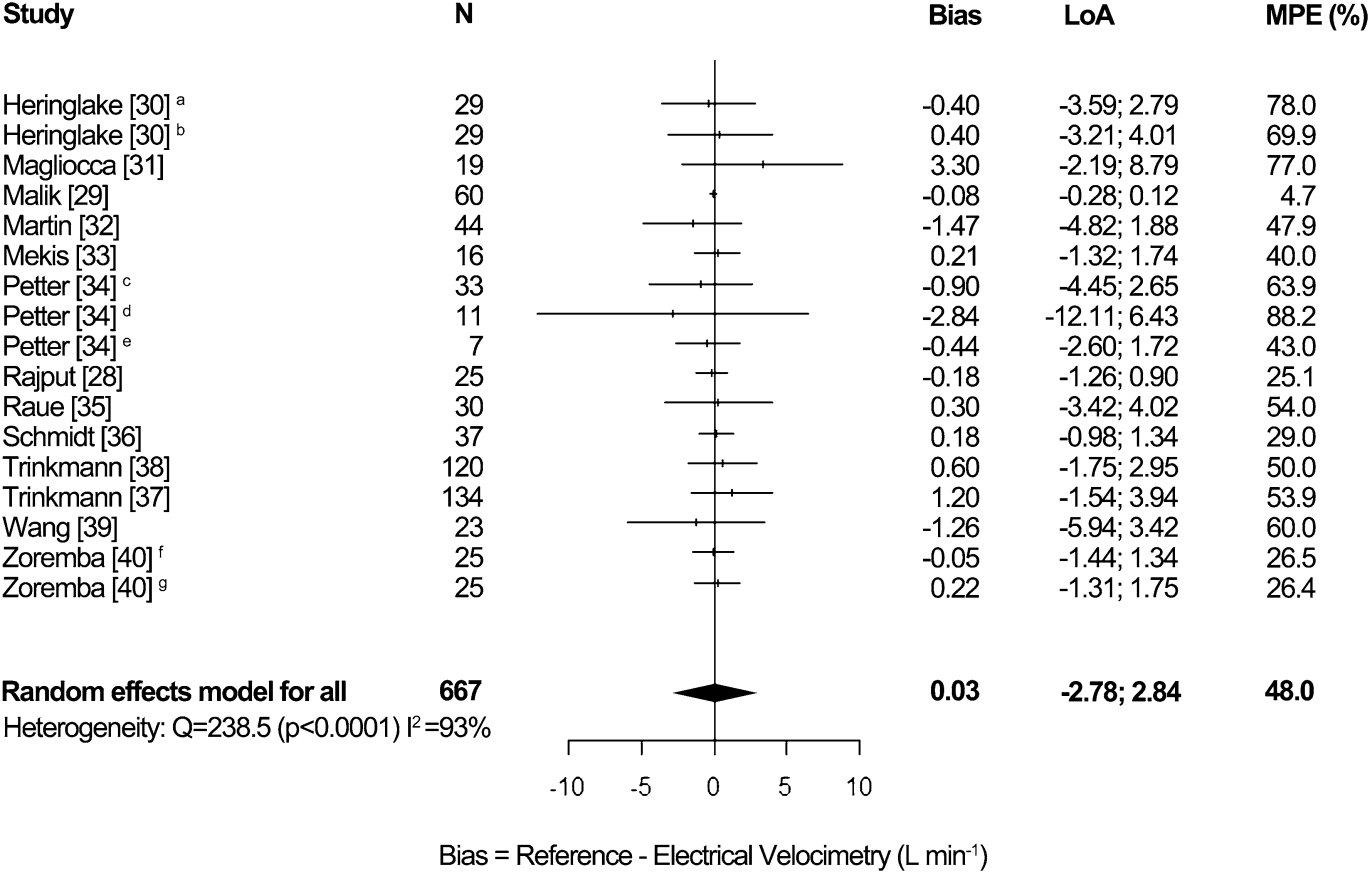
Forest plot showing the bias, LoA and MPE for the studies in adults. The random effects pooled bias was 0.03 L min^−1^, LoA − 2.78 to 2.84 L min^−1^ and MPE 48.0%. Significant heterogeneity was detected (I^2^ = 93%, p < 0.0001). ^a^OR, ^b^ICU, ^c^at rest, ^d^during exercise, ^e^during NO inhalation, ^f^intermittent PAC as reference method, ^g^TPTD as reference method, *LoA* limits of agreement, *MPE* mean percentage error, *N* number of patients

The pooled results for the adult studies are shown in Fig. 3. The overall random effects pooled bias was 0.03 L min^−1^ [95% CI − 0.23; 0.29], LoA − 2.78 to 2.84 L min^−1^ and MPE 48.0%. Inter-study heterogeneity was high (I^2^ = 93%, p < 0.0001). For two studies multiple data for a patient is presented in two or three different rows in the forest plot, as those studies presented multiple outcome measures for different clinical circumstances [30, 34]. Therefore, the number of patients in the forest plot for adults (N = 667) differs from the actual number of adult patients (N = 620).

### Subgroup analyses, adults

Figure 7 in Appendix 7 shows a subgroup analysis for reference method in adults. The subgroup intermittent TD showed a random effects pooled bias of 0.04 L min^−1^ [95% CI − 0.28; 0.37], LoA − 3.14 to 3.22 L min^−1^ and MPE 53.5%. Heterogeneity was high (I^2^ = 80%, p < 0.0001). The subgroup continuous TD showed a pooled bias of − 0.56 L min^−1^ [95% CI − 1.70; 0.57], LoA − 2.90 to 1.78 L min^−1^ and MPE 31.1%. Heterogeneity was high (I^2^ = 82%, p = 0.02). The subgroup other reference showed a pooled bias of 0.16 L min^−1^ [95% CI − 0.57; 0.90], LoA − 2.34 to 2.66 L min^−1^ and MPE 48.5%. Heterogeneity was high (I^2^ = 97%, p < 0.0001). There was no statistically significant difference in subgroup effects (p = 0.55).

Figure 8 in Appendix 8 shows a subgroup analysis for clinical setting in adults. The subgroup cardiac surgery showed a random effects pooled bias of 0.01 L min^−1^ [95% CI − 0.14; 0.17], LoA − 1.34; 1.36 L min^−1^ and MPE 33.3%. Heterogeneity was high (I^2^ = 73%, p < 0.01). The subgroup OR showed a pooled bias of 1.00 L min¯^1^ [95% CI − 3.47; 5.47], LoA − 4.05; 6.05 L min^−1^ and MPE 67.7%. Heterogeneity was high (I^2^ = 97%, p < 0.0001). The subgroup ICU showed a pooled bias of 0.04 L min^−1^ [95% CI − 0.18; 0.27], LoA − 2.37; 2.45 L min^−1^ and MPE 42.9%. Heterogeneity was moderate (I^2^ = 38%, p = 0.17). The subgroup other clinical setting showed a pooled bias of − 0.35 L min^−1^ [95% CI − 1.22; 0.53], LoA − 3.17; 2.47 L min^−1^ and MPE 53.5%. Heterogeneity was high (I^2^ = 96%, p < 0.0001). There was no statistically significant difference in subgroup effects (p = 0.82). The study by Mekis et al. was conducted during cardiac surgery and in the ICU [33]. Therefore, we divided the data of this study in three rows, namely before and immediately post cardiac surgery and in the ICU. As three rows in the subgroup analysis for clinical setting replace one row in the forest plot for adults (Fig. 3), the number of patients and pooled data presented in the subgroup analysis for clinical setting slightly differ from the actual pooled data presented in the forest plot for adults.

### Synthesis of results, pediatrics

**Fig. 4:**
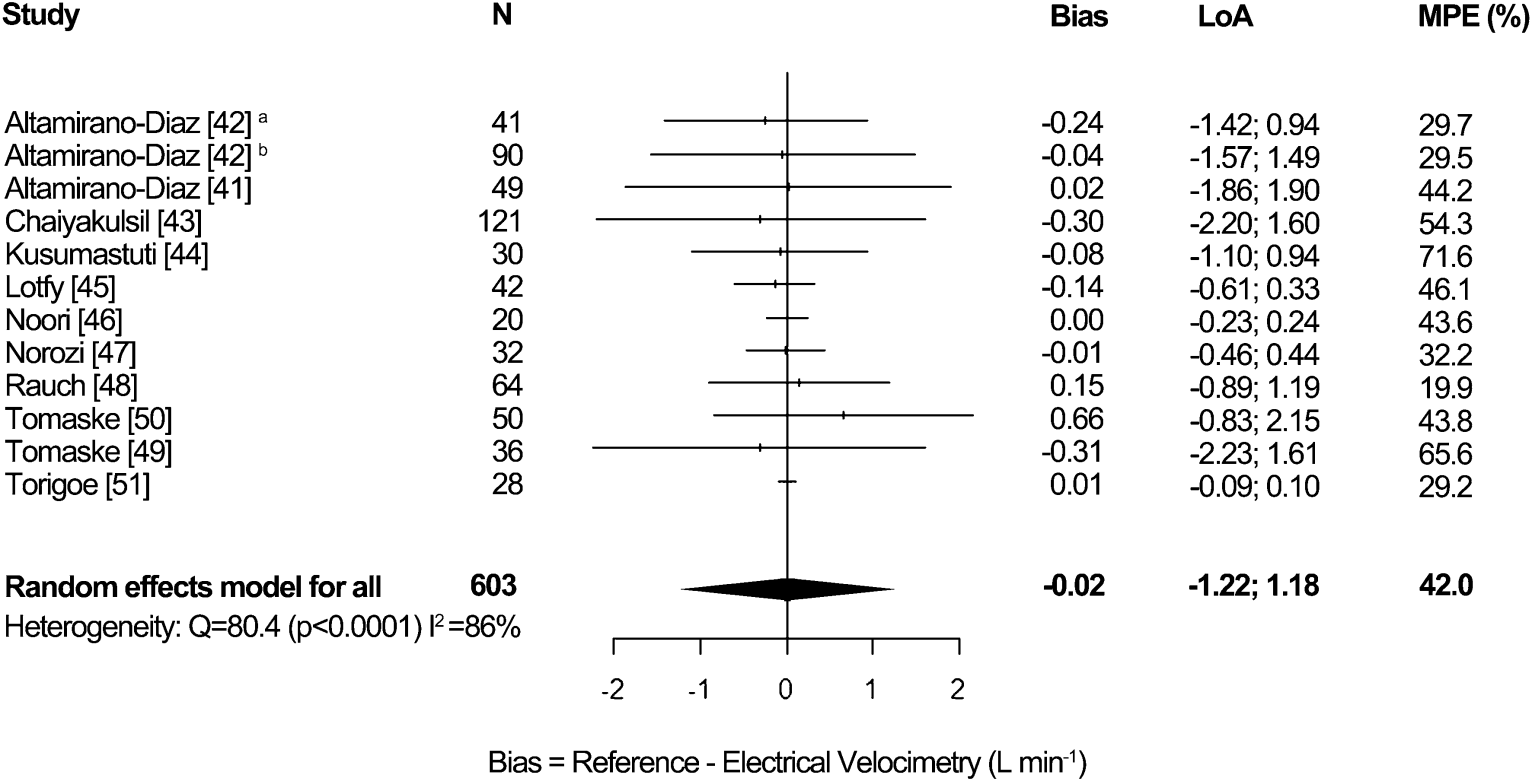
Forest plot showing the bias, LoA and MPE for the studies in pediatrics. The random effects pooled bias was − 0.02 L min^−1^, LoA − 1.22 to 1.18 L min^−1^ and MPE 42.0%. Significant heterogeneity was detected (I^2^ = 86%, p < 0.0001). ^a^normal weight, ^b^overweight and obese, *LoA* limits of agreement, *MPE* mean percentage error, *N* number of patients

Figure 4 demonstrates the pooled results for the pediatric studies. The overall random effects pooled bias was − 0.02 L min^−1^ [95% CI − 0.09; 0.05], LoA − 1.22 to 1.18 L min^−1^ and MPE 42.0%. Heterogeneity was high (I^2^ = 86%, p < 0.0001).

### Subgroup analysis, pediatrics

Figure 9 in Appendix 9 shows a subgroup analysis for reference method in pediatrics. The subgroup TTE in children showed a random effects pooled bias of − 0.10 L min^−1^ [95% CI − 0.25, 0.04], LoA − 1.61 to 1.41 L min^−1^ and MPE 43.9%. Heterogeneity was high (I^2^ = 75%, p < 0.001). The subgroup TTE in neonates showed a pooled bias of 0.01 L min¯^1^ [95% CI − 0.01, 0.02], LoA − 0.14 to 0.16 L min^−1^ and MPE 35.1%. No heterogeneity was detected (I^2^ = 0%, p = 0.94).The subgroup other reference method in children showed a pooled bias of 0.15 L min^−1^ [95% CI − 0.14; 0.44], LoA − 0.73 to 1.03 L min^−1^ and MPE 41.6%. Heterogeneity was high (I^2^ = 96%, p < 0.0001). There was no statistically significant difference in subgroup effects (p = 0.21).

### Risk of publication bias across studies

**Fig. 5:**
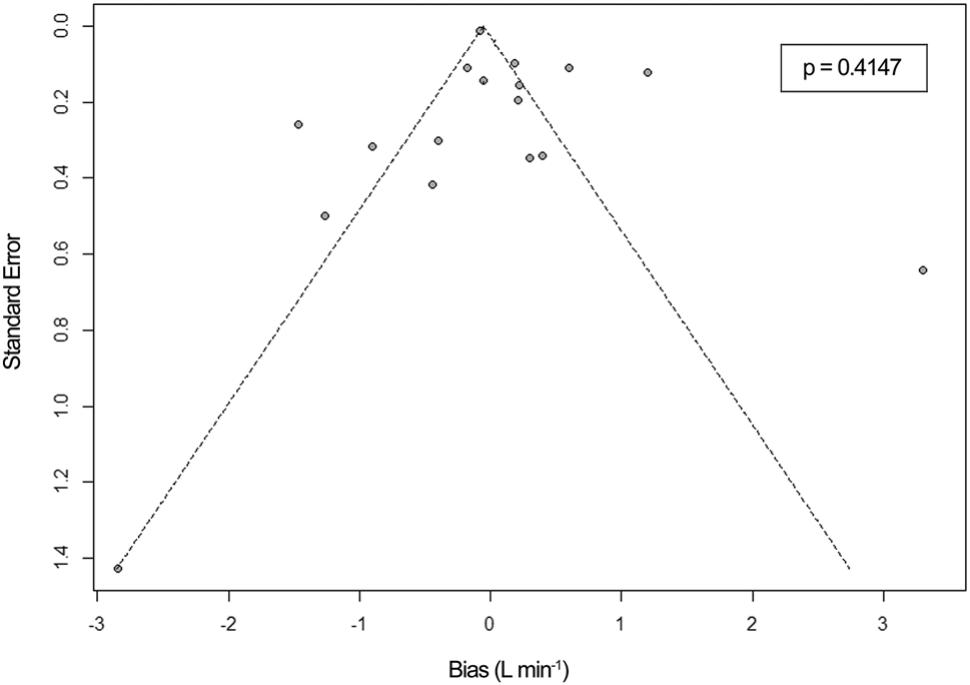
Funnel plot for detection of publication bias across included studies in adults. Egger’s regression test showed no significant p-value (p = 0.4147). The funnel plot shows asymmetry

**Fig. 6:**
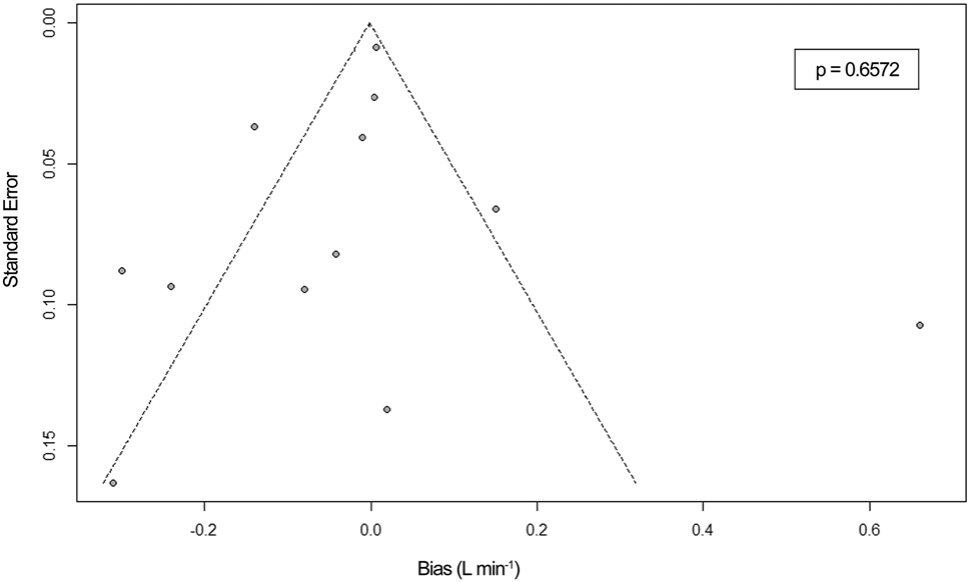
Funnel plot for detection of publication bias across included studies in pediatrics. Egger’s regression test showed no significant p-value (p = 0.6572). The funnel plot shows asymmetry

To detect risk of bias across studies, funnel plots are shown in Figs. 5 and 6. Egger’s regression test showed no significant *p* value for both adults (p = 0.4147) and pediatrics (p = 0.6572), indicating a low risk of publication bias [27]. However, for both groups asymmetry could be detected, which could be caused by publication bias or high heterogeneity. The latter is most likely the explanation. However, publication bias cannot be excluded.

### Trending ability

Seven of the thirteen studies in adults assessed trending ability, applying several statistical analyses [28–31, 33, 34, 39]. Magliocca et al. and Wang et al. analysed trending ability using a 4-quadrant plot, showing a concordance rate of respectively 100% and 56.5% [31, 39]. Other statistical methods were a time plot [29], a receiver operator characteristic curve [28], descriptive analyses of changes in CO for the whole study population [33, 34] or individuals [30]. None of the studies in pediatrics evaluated trending. Due to a lack of agreement on the statistical methodology, no pooled results can be calculated.

## Discussion

### Summary of evidence

This meta-analysis of 24 studies, which assesses the accuracy and precision of EC, shows a pooled bias of 0.03 L min¯^1^ [95% CI − 0.23, 0.29], LoA − 2.78 to 2.84 L min^−1^ and MPE 48.0% in adult studies. In pediatric studies pooled bias was − 0.02 L min^−1^ [95% CI − 0.09; 0.05], LoA − 1.22 to 1.18 L min^−1^ and MPE 42.0%. Inter-study heterogeneity was high in both adults (I^2^ = 93%, p < 0.0001) and pediatrics (I^2^ = 86%, p < 0.0001).

Although the pooled bias in both adult and pediatric studies was close to zero, high accuracy cannot be assumed, as the range of the bias in the studies was wide. The direction of the bias (positive or negative) is inconsistent and cannot be predicted in the clinical setting, which corresponds with the high inter-study heterogeneity. Pooled MPE in all subgroups were above the recommended 30% [20]. Therefore, EC cannot replace TD and TTE for the measurement of absolute CO values.

The ICON^®^ and Aesculon^®^ monitors were included in three other meta-analyses [10–12]. Importantly, the data of the three other meta-analyses are the result of subgroup analyses for TEB, including EC but also other devices based on other algorithms. Therefore, no conclusions may be drawn for EC only. Peyton and Chong found a bias of − 0.10 L min^−1^ and a MPE of 42.9% in adults, by a subgroup analysis including five EC studies and seven studies based on other algorithms [12]. Joosten et al. performed a subgroup analysis including four EC studies and six studies based on other algorithms and found a bias of − 0.22 L min^−1^ and a MPE of 42% in adults [10]. These results are comparable with our findings. Suehiro et al. found a bias of − 0.03 L min^−1^ and a MPE of 23.6% in pediatrics, by a subgroup analysis of four EC studies and four studies based on other algorithms [11]. We found similar bias, but could not confirm the low MPE. In contrast to above mentioned reviews, our results are derived from EC studies only. Furthermore, subgroup analyses of the gold standard in adults (TD) and most commonly used technique in pediatrics (TTE) were applied in our meta-analysis. This leads to definitive validation of EC compared to these methods. Besides, our meta-analysis includes more studies, and therefore more patients and more clinical settings than previous meta-analyses. So in numbers and diversity our study contributes and elaborates on the topic.

When compared to other minimally or non-invasive techniques used in clinical practice, most devices show a MPE of more than 30% [10, 12, 68–76]. Therefore, Peyton and Chong have suggested to change the acceptable MPE to 45%, ensuring a higher rate of agreement in new methods [12]. MPE is determined by the reference and tested method and highly influenced by the clinical condition. The lowest bias and MPE are found in validation studies during cardiac surgery [68, 77, 78]. The worst results are found during sepsis and septic shock as the bias of most non-invasive devices is negatively influenced by a low systemic vascular resistance (SVR) [68, 74, 75, 79–81]. Which device should be the reference method and under which clinical condition the validation needs to be performed, remains subject of discussion.

**Fig. 7:**
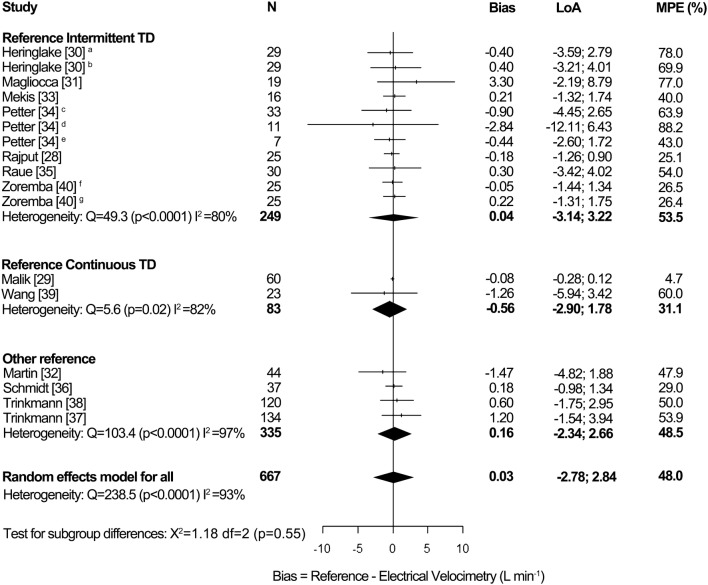
Forest plot showing the results of subgroup analysis for reference method in adults. The subgroup intermittent TD showed a random effects pooled bias of 0.04 L min^−1^ [95% CI − 0.28; 0.37], LoA − 3.14 to 3.22 L min^−1^ and MPE 53.5%. Heterogeneity was high (I^2^ = 80%, p < 0.0001). The subgroup continuous TD showed a pooled bias of − 0.56 L min^−1^ [95% CI − 1.70; 0.57], LoA − 2.90 to 1.78 L min^−1^ and MPE 31.1%. Heterogeneity was high (I^2^ = 82%, p = 0.02). The subgroup other reference showed a pooled bias of 0.16 L min^−1^ [95% CI − 0.57; 0.90], LoA − 2.34 to 2.66 L min^−1^ and MPE 48.5%. Heterogeneity was high (I^2^ = 97%, p < 0.0001). There was no statistically significant difference in subgroup effects (p = 0.55). ^a^OR, ^b^ICU, ^c^at rest, ^d^during exercise, ^e^during NO inhalation, ^f^intermittent PAC as reference method, ^g^TPTD as reference method. *LoA* limits of agreement, *MPE* mean percentage error, *N* number of patients, *TD* thermodilution

The subgroup analysis for reference method in adults (Fig. 7 in Appendix 7) showed a relatively high MPE (53.5%) for intermittent TD and a relatively low MPE (31.1%) for continuous TD. The high MPE for intermittent TD can be explained by the high MPE of the included studies. As the subgroup continuous TD consists of only two studies, the low MPE can be explained by the extremely low MPE (4.7%) of one included study [29].

**Fig. 8:**
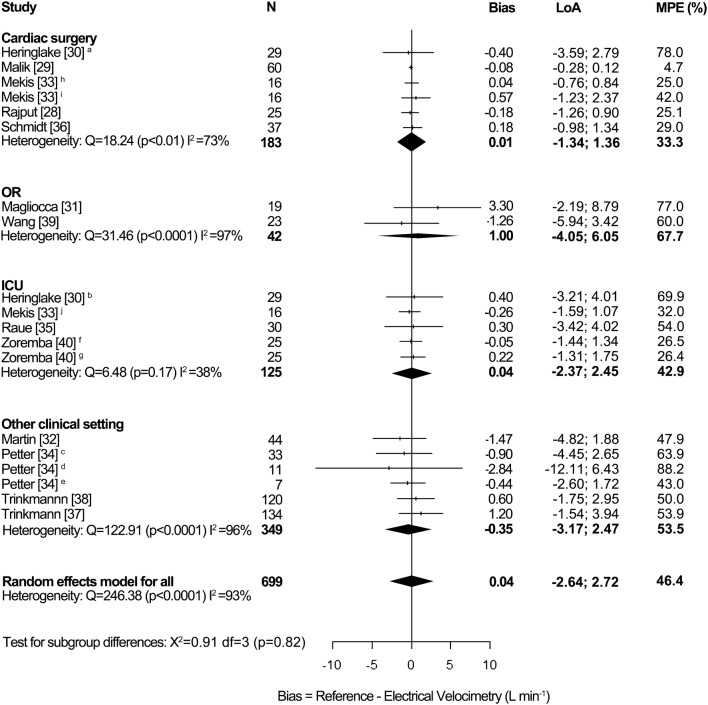
Forest plot showing the results of subgroup analysis for clinical setting in adults. The subgroup cardiac surgery showed a random effects pooled bias of 0.01 L min^−1^, LoA − 1.34; 1.36 L min^−1^ and MPE 33.3%. Heterogeneity was high (I^2^ = 73%, p < 0.01). The subgroup OR showed a pooled bias of 1.00 L min¯^1^, LoA − 4.05; 6.05 L min^−1^ and MPE 67.7%. Heterogeneity was high (I^2^ = 97%, p < 0.0001). The subgroup ICU showed a pooled bias of 0.04 L min^−1^, LoA − 2.37; 2.45 L min^−1^ and MPE 42.9%. Heterogeneity was moderate (I^2^ = 38%, p = 0.17). The subgroup other clinical setting showed a pooled bias of − 0.35 L min^−1^, LoA − 3.17; 2.47 L min^−1^ and MPE 53.5%. Heterogeneity was high (I^2^ = 96%, p < 0.0001). There was no statistically significant difference in subgroup effects (p = 0.82). ^a^OR, ^b^ICU, ^c^at rest, ^d^during exercise, ^e^during NO inhalation, ^f^intermittent PAC as reference method, ^g^TPTD as reference method, ^h^before cardiac surgery, ^i^immediately post cardiac surgery, ^j^ICU. *ICU* intensive care unit, *LoA* limits of agreement, *MPE* mean percentage error, *N* number of patients, *OR* operation room

The subgroup analysis for clinical setting in adults (Fig. 8 in Appendix 8) showed a low bias (0.01 L min^−1^) and a relatively low MPE (33.3%) during cardiac surgery, probably due to the hypodynamic status with low CO and high SVR. The studies in this subgroup showed a mean CO of 4.1 ± 0.2 L min^−1^. The other included adult studies showed a statistical higher (p < 0.05) mean CO of 6.3 ± 1.7 L min^−1^. The OR subgroup, consisting of two studies during liver transplantation [31, 39], showed a relatively high bias (1.00 L min^−1^) and high MPE (67.7%), this could be explained by the hyperdynamic status (high CO and low SVR) which is often seen during these procedures [31, 68]. The patient characteristics in the ICU subgroup differed too much to draw conclusions for this subgroup, as it concerned post cardiac surgery patients [30, 33], patients suffering from systemic inflammatory response syndrome or sepsis post-surgery [35] or critically ill patients post-surgery [40] (Table 1). The same accounts for the studies included in the other clinical setting subgroup, which concerned pregnant women [32], hemodynamically stable cardiac patients [37, 38] or took partly place during exercise or NO inhalation [34] (Table 1).

**Fig. 9:**
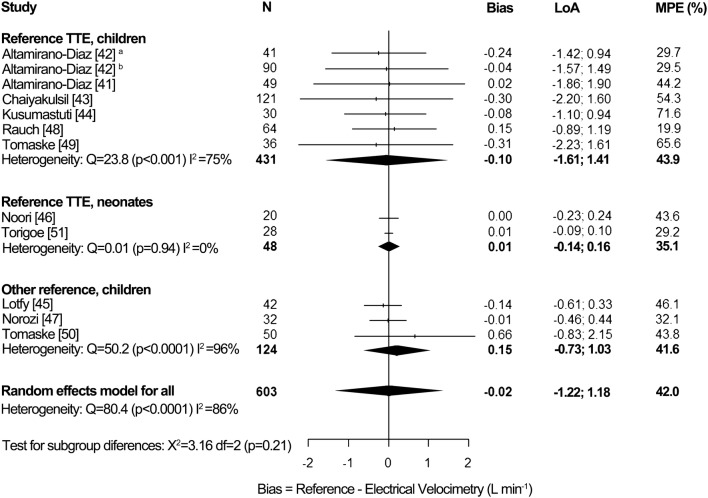
Forest plot showing the results of subgroup analysis for reference method in pediatrics. The subgroup TTE in children showed a random effects pooled bias of − 0.10 L min^−1^, LoA − 1.61 to 1.41 L min^−1^ and MPE 43.9%. Heterogeneity was high (I^2^ = 75%, p < 0.001). The subgroup TTE in neonates showed a pooled bias of 0.01 L min¯^1^, LoA − 0.14 to 0.16 L min^−1^ and MPE 35.1%. No heterogeneity was detected (I^2^ = 0%, p = 0.94).The subgroup other reference method in children showed a pooled bias of 0.15 L min^−1^, LoA − 0.73 to 1.03 L min^−1^ and MPE 41.6%. Heterogeneity was high (I^2^ = 96%, p < 0.0001). There was no statistically significant difference in subgroup effects (p = 0.21). ^a^normal weight, ^b^overweight and obese. *LoA* limits of agreement, *MPE* mean percentage error, *N* number of patients, *TTE* transthoracic echocardiography

The results for the subgroup TTE children were comparable to the pooled results for pediatric studies. The subgroup TTE neonates showed a relatively low MPE (35.1%) (Fig. 9 in Appendix 9).

Although a subgroup analysis for clinical setting in adults was performed post hoc, we decided not to perform the same subgroup analysis in pediatric studies, as the clinical settings differed too much (Table 2), which should lead to very small subgroups. No subgroup analyses for age were performed, as the age ranged too much in the individual adult and pediatric studies (Tables 1, 2).

### Recommendations for clinicians

EC cannot replace TD and TTE for the measurement of absolute CO values. However, as the MPE is comparable to clinically used minimally or non-invasive hemodynamic monitors, EC could complement monitoring in the ICU and NICU, providing continuous monitoring, relevant for goal-directed therapy and clinical decision-making. This should be further investigated. In the OR, monopolar electrocauterization interferes with the EC measurement [82]. Bipolar electrocauterization does not.

### Limitations

This study has multiple limitations. Firstly, population selection bias could be present. Most studies took place in cardiac surgical setting [28–30, 33, 36, 44]. Although hemodynamic instability can be present, cardiac surgery is characterized by low CO and high SVR [68, 77, 78], which could be an explanation for the low bias and relatively low MPE in the cardiac surgery subgroup. The low bias and MPE influence the pooled data in adults.

Another limitation is the LoA and MPE as outcome measures. Both are influenced by the error of the reference method. All reference methods have their own inherent error and do not provide an accurate and precise measurement of CO. For example, the precision of different TD devices is proved to be 13% by Stetz et al. [83]. Slagt et al. showed a precision of 6.7% for TPTD [81]. For intermittent PAC, precisions of 6.4% [84], 8.4% [85] and 16.2% [28] are described. For TTE, Mercado et al. showed a 9% precision [86] and we derived 8.4% precision based on the data by Tomaske et al. (See Table 7 in Appendix 4) [49]. Concerning TEE, precisions of 12.8% [84] and 16.0% [85] are described. For Fick method, a precision of 27.4% was calculated from the data by Trinkmann et al. (See Table 6 in Appendix 3) [38]. Critchley and Critchley proved that the MPE depends on both the precision of the reference and tested method, according to the following equation [20]:

7 $$MPE = \sqrt {\left[ {\left( {precision_{reference} } \right)^{2} + \left( {precision_{test} } \right)^{2} } \right]} .$$

To draw conclusions from the MPE concerning the precision of the tested method, Cecconi recommends to measure the precision of the reference method within the study using repeated measurements and according to the following equation:

8 $$Precision\,method\,x = \frac{1.96*SD\,of\,reproductability}{mean\,CO\,method\,x}.$$

The precision of the tested method can then be calculated, according to Eq. (7) [25]. Hapfelmeier proved that Eq. (7) is not completely true, as the overall precision and MPE depend on the method’s variability about the true values as well [87]. In spite of its inaccuracy, Eq. (7) indicates that the precision of both reference and tested method influence the MPE and should therefore be calculated for proper interpretation of the LoA and MPE. Only a few studies measured both (Tables 3, 4) [28, 38, 40, 49].

In addition to the latter described limitation, the different reference methods should be described as another limitation. It is questionable whether the included studies, based on different reference methods, are comparable. This could be an explanation for the high inter-study heterogeneity found in our review. Therefore, we applied subgroup analyses of the gold standard TD in adults and most commonly used technique TTE in pediatrics. The results of the subgroup analyses are discussed earlier. Inter-study heterogeneity decreased, but remained high. The subgroup TTE in neonates showed no heterogeneity (I^2^ = 0%), as the two included studies showed comparable results.

To assess the statistical analysis in the included studies, we developed an additional domain for the modified QUADAS-2 tool. This has not been done previously. The risk of bias in individual studies was high in the statistical analysis domain (Tables 3, 4), which is a limitation of this review too. First, in some studies, the direction of the bias was unclear [28, 29, 40, 44, 47]. Second, the SD described in the manuscript did not correspond with the LoA in the figure [28, 29, 43]. Third, the recalculated MPE differed from the value presented in five studies [29, 37, 43, 44, 50]. For those studies, the differences in MPE (defined as recalculated MPE—presented MPE) were 1.1% [29], 2.9% [37], 26.8% [43], 58.4% [44], − 5.1% [50] (See Tables 6, 7 in Appendix 3, 4). In many cases, the MPE could not be recalculated [30, 31, 33–35, 38, 40, 41, 45, 47–49]. Fourth, the Bland–Altman analysis may only be applied for independent observations. In case of multiple observations per individual and in the absence of major hemodynamic changes, a modification of the Bland–Altman analysis for repeated measurements should be applied [88–90]. Many of the included studies used multiple observations per individual, but did not apply the modified Bland–Altman analysis [28, 32–34, 37–39, 43, 45, 50, 51]. This can lead to narrower LoA and a lower MPE in the individual studies [88, 89]. Lastly, only a few studies assessed the precision of both reference and tested method [28, 38, 40, 49], which is discussed earlier. Overall, the high risk of bias in the statistical domain causes the pooled data in this review to be less reliable.

Besides, for two adult studies multiple data for a patient is presented in two or three different rows in the forest plot, as those studies presented multiple outcome measures for different clinical circumstances [30, 34]. As the clinical conditions of both measurement points are different, the data can be considered as independent. Therefore it is statistically justified to assess these data separate.

Furthermore, some studies were excluded from our meta-analysis because of assessment of cardiac index, stroke volume or CO presented as mL kg^−1^ min^−1^, instead of CO as L min^−1^ [52–56, 58–60, 62–66]. These studies could have been a contribution to our results.

### Trending ability

Monitoring changes in CO is relevant in clinical practice to measure the effect of an intervention. Despite its inability to measure absolute CO values, which is assessed by the Bland–Altman analysis, EC could still be applicable as trend monitor. To achieve acceptable trending ability, good precision is required, independent of the accuracy [91]. For the assessment of trending ability different methods are described, of which the for-quadrant plot and the polar plot are recommended [92–94]. Seven of the thirteen studies in adults assessed trending ability, applying several statistical analyses [28–31, 33, 34, 39]. None of the studies in pediatrics evaluated trending. Due to a lack of agreement on the statistical methodology, it is difficult to compare results and draw conclusions, which is a limitation of this review.

### Future research

Our study focuses on the ICON^®^/Aesculon^®^ monitor for evaluating EC. The ICON^®^/Aesculon^®^ monitor is a device in development and future research should clarify its place between existing hemodynamic monitoring devices. The high risk of bias in the statistical analysis domain of the modified QUADAS-2 tool emphasizes the lack of consensus how to present data in validation studies, despite the fact that good proposals have been published [20, 25, 87, 91]. Consensus is required to interpret results of different studies and draw conclusions. Future validation studies with regard to EC, should also focus on trending ability [92–94]. Combined with studies on the applicability of EC for continuous CO monitoring and goal-directed therapy, this will provide useful clinical advice.

## Conclusion

This meta-analysis of 24 studies, which assesses the accuracy and precision of non-invasive CO measurement by EC compared to a reference method, shows a pooled bias of 0.03 Lmin¯^1^ [95% CI − 0.23; 0.29], LoA − 2.78 to 2.84 L min^−1^ and MPE was 48.0% in adult studies. In pediatric studies the pooled bias was − 0.02 L min^−1^ [95% CI − 0.09; 0.05], LoA − 1.22 to 1.18 L min^−1^ and MPE 42.0%. Inter-study heterogeneity was high for both adults (I^2^ = 93%, p < 0.0001) and pediatrics (I^2^ = 86%, p < 0.0001). Despite the low bias in both adults and pediatrics, the pooled MPE were above the recommended 30%. Therefore, EC cannot replace TD and TTE for the measurement of absolute CO values. The trending ability of EC could not be assessed in this meta-analysis, due to a lack of agreement on the statistical methodology in the included studies. So, EC might still be applicable as a trend monitor to measure acute changes in CO, which is relevant for clinical decision-making. This should be an important part of future research, especially as EC is safe and easy to apply.

## Appendix 1

See Table 5.

**Table 5:** PRISMA checklist, reproduced from Liberati et al. [21]

PRISMA checklist		
Title		
Title	1.	Identify the report as a systematic review, meta-analysis, or both
Abstract		
Structured summery	2.	Provide a structured summary including, as applicable: background; objectives; data sources; study eligibility criteria, participants, and interventions; study appraisal and synthesis methods; results; limitations; conclusions and implications of key findings; systematic review registration number
Introduction		
Rationale	3.	Describe the rationale for the review in the context of what is already known
Objectives	4.	Provide an explicit statement of questions being addressed with reference to participants, interventions, comparisons, outcomes, and study design (PICOS)
Methods		
Protocol and registration	5.	Indicate if a review protocol exists, if and where it can be accessed (e.g., Web address), and, if available, provide registration information including registration number
Eligibility criteria	6.	Specify study characteristics (e.g., PICOS, length of follow-up) and report characteristics (e.g., years considered, language, publication status) used as criteria for eligibility, giving rationale
Information sources	7.	Describe all information sources (e.g., databases with dates of coverage, contact with study authors to identify additional studies) in the search and date last searched
Search	8.	Present full electronic search strategy for at least one database, including any limits used, such that it could be repeated
Study selection	9.	State the process for selecting studies (i.e., screening, eligibility, included in systematic review, and, if applicable, included in the meta-analysis)
Data collection process	10.	Describe method of data extraction from reports (e.g., piloted forms, independently, in duplicate) and any processes for obtaining and confirming data from investigators
Data items	11.	List and define all variables for which data were sought (e.g., PICOS, funding sources) and any assumptions and simplifications made
Risk of bias in individual studies	12.	Describe methods used for assessing risk of bias of individual studies (including specification of whether this was done at the study or outcome level), and how this information is to be used in any data synthesis
Summary measures	13.	State the principal summary measures (e.g., risk ratio, difference in means)
Synthesis of results	14.	Describe the methods of handling data and combining results of studies, if done, including measures of consistency (e.g., I^2^) for each meta-analysis
Risk of bias across studies	15.	Specify any assessment of risk of bias that may affect the cumulative evidence (e.g., publication bias, selective reporting within studies)
Additional analyses	16.	Describe methods of additional analyses (e.g., sensitivity or subgroup analyses, meta-regression), if done, indicating which were pre-specified
Results		
Study selection	17.	Give numbers of studies screened, assessed for eligibility, and included in the review, with reasons for exclusions at each stage, ideally with a flow diagram
Study characteristics	18.	For each study, present characteristics for which data were extracted (e.g., study size, PICOS, follow-up period) and provide the citations
Risk of bias within studies	19.	Present data on risk of bias of each study and, if available, any outcome level assessment (see item 12)
Results of individual studies	20.	For all outcomes considered (benefits or harms), present, for each study: (a) simple summary data for each intervention group (b) effect estimates and confidence intervals, ideally with a forest plot
Synthesis of results	21.	Present results of each meta-analysis done, including confidence intervals and measures of consistency
Risk of bias across studies	22.	Present results of any assessment of risk of bias across studies (see Item 15)
Additional analysis	23.	Give results of additional analyses, if done (e.g., sensitivity or subgroup analyses, meta-regression [see Item 16])
Discussion		
Summary of evidence	24.	Summarize the main findings including the strength of evidence for each main outcome; consider their relevance to key groups (e.g., healthcare providers, users, and policy makers)
Limitations	25.	Discuss limitations at study and outcome level (e.g., risk of bias), and at review-level (e.g., incomplete retrieval of identified research, reporting bias)
Conclusions	26.	Provide a general interpretation of the results in the context of other evidence, and implications for future research
Funding		
Funding	27.	Describe sources of funding for the systematic review and other support (e.g., supply of data); role of funders for the systematic review

## Appendix 2: Search strategy in PubMed

All fields:

1. Measure OR measured OR measurement OR measurements OR measures OR measuring
2. Monitor OR monitored OR monitors OR monitoring
3. Determine OR determined OR determines OR determination OR determinations OR determining
4. Parameter OR parameters
5. #1 OR #2 OR #3 OR #4
6. Hemodynamic OR hemodynamics OR haemodynamic OR haemodynamics
7. #5 AND #6
8. Cardiac output OR Heart output OR Cardiac Index OR Stroke volume
9. #7 OR #8
10. Electrical velocimetry OR electrical velocimeter
11. Electrical cardiometry OR electrical cardiometer
12. Aesculon
13. ICON
14. #10 OR #11 OR #12 OR #13
15. #9 AND #14

## Appendix 3

See Table 6.

**Table 6:** Data of included adult studies

Author	Tested method	Reference method	Mean CO^a^ (L min^−1^)	COrange^b^ (L min^−1^)	Bias (Ref – Test) (L min^−1^)	Precision^c^ (L min^−1^)	LoA (L min^−1^)	MPE^d^ (%)	Precision Test^e^ (%)	Precision Ref^e^ (%)	Trending Ability, CR (%)	Patients	Data
Heringlake [30]^f^	Aesculon	PACi	**4.1**	*2.9*–*6.2*	− 0.40	**1.6**	− 3.6; 2.8	**78.0**	NA	NA	NA	29	29
Heringlake [30]^g^	Aesculon	PACi	**5.2**	*3.4*–*7.4*	0.40	**1.8**	− 3.2; 4.0	**69.9**	NA	NA	NA	29	29
Magliocca [31]	ICON	PACi	*7.1*	*3.5*–*11.6*	3.30	2.8	− 2.2; 8.8	77.0	NA	NA	100	19	95
Malik [29]	ICON	PACc	**4.2**	*2.3*–*5.5*	− 0.08	(0.15) **0.10**	− 0.3; 0.1	(3.6) **4.7**	NA	NA	NA	60	180
Martin [32]	ICON	TTE	**7.0**	NA	− 1.47	**1.7**	− 4.8; 1.9	47.9	NA	NA	NA	44	44
Mekis [33]^h^	Aesculon	PACi	*3.8*	*2.3*–*7.5*	0.21	0.8	− 1.3; 1.7	40.0	NA	NA	NA	16	150
Mekis [33]^i^	Aesculon	PACi	*3.2*	*2.3*–*5.0*	0.04	0.4	− 0.8; 0.8	25.0	NA	NA	NA	16	57
Mekis [33]^j^	Aesculon	PACi	*4.3*	*2.5*–*6.6*	0.57	0.9	− 1.2; 2.4	42.0	NA	NA	NA	16	64
Mekis [33]^k^	Aesculon	PACi	*4.2*	*2.7*–*7.5*	− 0.26	0.7	− 1.6; 1.1	32.0	NA	NA	NA	16	29
Petter [34]^l^	Aesculon	PACi	*5.6*	*3.8*–*10.5*	− 0.90	1.8	− 4.5; 2.7	63.9	NA	NA	NA	33	33
Petter [34]^m^	Aesculon	PACi	*10.5*	*3.9*–*22.5*	− 2.84	4.7	− 12.1; 6.4	88.2	NA	NA	NA	11	11
Petter [34]^n^	Aesculon	PACi	*5.0*	*4.2*–*6.8*	− 0.44	1.1	− 2.6; 1.7	43.0	NA	NA	NA	7	7
Rajput [28]	ICON	PACi	**4.3**	*1.9*–*6.9*	− 0.18	(0.50) **0.55**	− 1.3; 0.9	**25.1**	19.6	16.2	NA	25	300
Raue [35]	Aesculon	TPTD	*6.9*	*5.0*–*11.9*	0.30	1.9	− 3.4; 4.0	54.0	NA	NA	NA	30	30
Schmidt [36]	Aesculon	TEE	**4.0**	*2.3*–*7.9*	0.18	0.6	− 1.0; 1.3	29.0	NA	NA	NA	37	37
Trinkmann [38]	Aesculon	Fick	**4.7**	*2.7*–*8.0*	0.60	1.2	− 1.8; 3.0	**50.0**	**13.4**	**27.4**	NA	120	120
Trinkmann [37]	Aesculon	MRI	***5.1***	*3.0*–*8.5*	1.2	1.4	− 1.5; 3.9	(51) **53.9**	15.0	NA	NA	134	134
Wang [39]	Aesculon	PACc	**7.8**	*3.3*–*12.5*	− 1.26	**2.4**	− 5.9; 3.4	60.0	NA	NA	56.5	23	207
Zoremba [40]^o^	Aesculon	PACi	*5.3*	*2.8*–*8.9*	− 0.05	0.7	− 1.4; 1.3	26.5	NA	NA	NA	25	25
Zoremba [40]^p^	Aesculon	TPTD	*5.8*	*2.8*–*10.0*	0.22	0.8	− 1.3; 1.8	26.4	NA	NA	NA	25	25

Values in brackets represent the data described in the original manuscript, which did not correspond with the data recalculated by the investigators

*CO* cardiac output, *CR* concordance rate, *LoA* limits of agreement, *MPE* mean percentage error, *MRI* magnetic resonance imaging, *NA* not available, *PACc* continuous pulmonary artery thermodilution, *PACi* intermittent pulmonary artery thermodilution, *Ref* reference method, *TEE* transesophageal echocardiography, *Test* tested method, *TPTD* transpulmonary thermodilution, *TTE* transthoracic echocardiography

^a^Bold data were calculated by investigators: $Mean\,CO = \frac{Mean\,COec + Mean\,COref}{2}$ . Italic data were calculated by investigators: $Mean\,CO = \frac{1.96 *SD}{MPE}$. Bold italicized data were derived from Bland–Altman plot, by reading and averaging the data points using a millimetre scale

^b^Italic data were derived from Bland–Altman plot, by reading the data points

^c^Bold data were calculated by investigators: $SD = \frac{upper\,LoA - lower\,LoA}{1.96 *2}$

^d^Bold data were calculated by investigators: $MPE = \frac{1.96*SD}{mean\,CO} * 100 \%$

^e^Bold data were calculated by investigators: $Precision\,method\,x = \frac{1.96*SD\,of\,reproducibility}{mean\,CO\,method x}$

^f^OR

^g^ICU

^h^Total

^i^Before cardiac surgery

^j^Immediately post cardiac surgery

^k^ICU

^l^At rest

^m^During exercise

^n^During NO inhalation

^o^Intermittent PAC as reference method

^p^TPTD as reference method

## Appendix 4

See Table 7.

**Table 7:** Data of included pediatric studies

Author	Tested method	Reference method	Mean CO^a^ (L min^−1^)	COrange^b^ (L min^−1^)	Bias^c^ (Ref – Test) (L min^−1^)	Precision^d^ (L min^−1^)	LoA (L min^−1^)	MPE^e^ (%)	Precision Test^f^ (%)	Precision Ref^f^ (%)	Trending Ability, CR (%)	Patients	Data
Altamirano-Diaz [42]^g^	ICON	TTE	4.0	*2.6*–*6.3*	− 0.24	0.6	− 1.4; 0.9	29.7	NA	NA	NA	41	41
Altamirano-Diaz [42]^h^	ICON	TTE	5.2	*2.3*–*8.5*	− 0.04	0.8	− 1.5; 1.5	29.5	NA	NA	NA	90	90
Altamirano-Diaz [41]	ICON	TTE	*4.3*	*2.5*–*6.1*	0.02	1.0	− 1.9; 1.9	44.2	NA	NA	NA	49	49
Chaiyakulsil [43]	ICON	TTE	**3.5**	NA	− 0.30	**1.0**	− 2.2; 1.6	(27.5) **54.3**	NA	NA	NA	121	121
Kusumastuti [44]	Aesculon	TTE	***1.4***	*0.3*–*3.3*	− 0.08	**0.5**	− 1.1; 0.9	(13.2) **71.6**	NA	NA	NA	30	30
Lotfy [45]	ICON	TEE	***1.02***	*0.4*–*1.7*	− 0.14	**0.2**	− 0.6; 0.3	**46.1**	NA	NA	NA	42	210
Noori [46]	Aesculon	TTE	0.5	*0.4*–*0.8*	0.00	0.1	− 0.2; 0.2	43.6	31.6	NA	NA	20	115
Norozi [47]	Aesculon	Fick	***1.4***	*0.3*–*3.9*	− ***0.01***	0.2	− 0.5; 0.4	**32.2**	NA	NA	NA	32	32
Rauch [48]	ICON	TTE	**5.2**	*3.3*–*8.5*	0.15	0.5	− 0.9; 1.2	**19.9**	NA	NA	NA	64	64
Tomaske [50]	Aesculon	PACi	**3.4**	*0.6*–*7.2*	0.66	**0.8**	− 0.8; 2.2	(48.9) **43.8**	NA	NA	NA	50	50
Tomaske [49]	Aesculon	TTE	***2.9***	*0.6*–*5.3*	− 0.31	**1.0**	− 2.2; 1.6	**65.6**	**9.41**	**8.43**	NA	36	36
Torigoe [51]	Aesculon	TTE	0.3	*0.1*–*0.7*	0.01	**0.0**	− 0.1; 0.1	29.2	NA	NA	NA	28	81

Values in brackets represent the data described in the original manuscript, which did not correspond with the data recalculated by the investigators

*CO* cardiac output, *CR* concordance rate, *LoA* limits of agreement, *MPE* mean percentage error, *NA* not available, *PACi* intermittent pulmonary artery thermodilution, *Ref* reference method, *TEE* transesophageal echocardiography, *Test* tested method, *TTE* transthoracic echocardiography

^a^Bold data were calculated by investigators: $Mean\,CO = \frac{Mean\,COec + Mean\,COref}{2}$. Italic data were calculated by investigators: $Mean\,Cardiac\,Output = \frac{1.96 *SD}{MPE}$. Bolditalic data were derived from Bland–Altman plot, by reading and averaging the data points using a millimetre scale

^b^Italic data were derived from Bland–Altman plot

^c^Bold italicized data were derived by contacting the author

^d^Bold data were calculated by investigators: $SD = \frac{upper\,LoA - lower\,LoA}{1.96 *2}$

^e^Bold data were calculated by investigators: $MPE = \frac{1.96 *SD}{mean\,CO} * 100 {\text{\% }}$

^f^Bold data were calculated by investigators: $Precision\,method\,x = 1.96 * Coefficient\,of\,Variation$

^g^Normal weight

^h^Overweight and obese

## Appendix 5

See Table 8.

**Table 8:** Modified QUADAS-2 tool, partly reproduced from Kim et al. [24]

**Modified QUADAS-2 assessment sheet**
Patient selection
Risk of bias: could the selection of patients have introduced bias?
1. Were subject population of interest and demographic data described?
2. Were inclusion and exclusion criteria clearly described?
Applicability: are there concerns that the included patients and setting do not match the review question?
Tested method
Risk of bias: could the conduct or interpretation of tested method for cardiac output monitoring have introduced bias?
3. Was the tested method described clearly? (calibration, position and characteristics of monitoring device)
Applicability: are there concerns that the tested method for cardiac output monitoring, it’s conduct or it’s interpretation differ from the review question?
Reference method
Risk of bias: could the reference method for cardiac output monitoring, it’s conduct or it’s interpretation have introduced bias?
4. Was the reference method described clearly? (calibration, position and characteristics of monitoring device)
5. Is the reference method likely to correctly measure cardiac output?
6. In case of TPTD or PAC: Was an average of three readings taken for analysis?
7. In case of echocardiography: Was the reference method assessed by the same, experienced investigator in each patient?
8. Were the reference method results interpreted without knowledge of the results of the tested method?
Applicability: are there concerns that the target condition as defined by the reference method for cardiac output monitoring does not match the review question?
Flow and timing
Risk of bias: could the analysis of flow and timing have introduced bias?
9. Were number of patients enrolled and who dropped out clearly described in the result?
10. Were the tested method and reference method measured simultaneously?
11. Was the method of acquiring paired measurement well described?
Statistical analysis
Risk of bias: could the statistical analysis have introduced bias?
12. Do the bias described in the manuscript and in the figures match? (that is, both are [tested method minus reference method] or [reference method minus tested method])
13. Do the SD described in the manuscript and the LoA in the figures match?
14. Does the mean percentage error described in the manuscript and recalculated by the reviewers match?
15. In case of multiple observations per individual, did they apply statistical analysis for repeated measurements?
16. Was the precision of the reference method measured within the study?
17. Was the precision of the tested method measured within the study?

Risk of bias is judged as “low,” “high, “unclear” or “not applicable”. If the answers to all signalling questions for a domain are “yes,” then risk of bias can be judged low. If any signalling question is answered “no,” potential for bias exists. The “unclear” category should be used only when insufficient data are reported to permit a judgment

## Appendix 6: List of excluded studies, after full-text analysis

1. Blohm ME, Hartwich J, Obrecht D, Kersten JF, Singer D (2017) Effect of patent ductus arteriosus and patent foramen ovale on left ventricular stroke volume measurement by electrical velocimetry in comparison to transthoracic echocardiography in neonates. Journal of clinical monitoring and computing 31 (3):589–598. 10.1007/s10877-016-9878-9
  Reason: Insufficient hemodynamic data (data presented as Stroke Volume)
2. Blohm ME, Obrecht D, Hartwich J, Mueller GC, Kersten JF, Weil J, Singer D (2014) Impedance cardiography (electrical velocimetry) and transthoracic echocardiography for non-invasive cardiac output monitoring in pediatric intensive care patients: a prospective single-center observational study. Critical care (London, England) 18 (6):603. 10.1186/s13054-014-0603-0
  Reason: Insufficient hemodynamic data (data presented as Stroke Volume)
3. Boet A, Jourdain G, Demontoux S, De Luca D (2016) Stroke volume and cardiac output evaluation by electrical cardiometry: accuracy and reference nomograms in hemodynamically stable preterm neonates. J Perinatol 36 (9):748–752. 10.1038/jp.2016.65
  Reason: No extractable Mean Percentage Error
4. Boet A, Jourdain G, Demontoux S, Hascoet S, Tissieres P, Rucker-Martin C, De Luca D (2017) Basic Hemodynamic Monitoring Using Ultrasound or Electrical Cardiometry During Transportation of Neonates and Infants. Pediatric critical care medicine : a journal of the Society of Critical Care Medicine and the World Federation of Pediatric Intensive and Critical Care Societies 18 (11):e488–e493. 10.1097/pcc.0000000000001298
  Reason: Insufficient hemodynamic data (data presented as Stroke Volume)
5. Borzage M, Heidari K, Chavez T, Seri I, Wood JC, Bluml S (2017) MEASURING STROKE VOLUME: IMPEDANCE CARDIOGRAPHY VS PHASE-CONTRAST MAGNETIC RESONANCE IMAGING. Am J Crit Care 26 (5):408–415. 10.4037/ajcc2017488
  Reason: Insufficient hemodynamic data (data presented as Stroke Volume)
6. Cox PBW, den Ouden AM, Theunissen M, Montenij LJ, Kessels AGH, Lance MD, Buhre W, Marcus MAE (2017) Accuracy, Precision, and Trending Ability of Electrical Cardiometry Cardiac Index versus Continuous Pulmonary Artery Thermodilution Method: A Prospective, Observational Study. Biomed Res Int 2017:2635151. 10.1155/2017/2635151
  Reason: Insufficient hemodynamic data (data presented as Cardiac Index)
7. Grollmuss O, Demontoux S, Capderou A, Serraf A, Belli E (2012) Electrical velocimetry as a tool for measuring cardiac output in small infants after heart surgery. Intensive care medicine 38 (6):1032-1039. 10.1007/s00134-012-2530-3
  Reason: Insufficient hemodynamic data (data presented as Stroke Volume)
8. 8. Grollmuss O, Gonzalez P (2014) Non-invasive cardiac output measurement in low and very low birth weight infants: a method comparison. Frontiers in pediatrics 2:16. 10.3389/fped.2014.00016
  Reason: Insufficient hemodynamic data (data presented as mL kg^−1^ min^−1^)
9. Hsu KH, Wu TW, Wu IH, Lai MY, Hsu SY, Huang HW, Mok TY, Lien R (2017) Electrical Cardiometry to Monitor Cardiac Output in Preterm Infants with Patent Ductus Arteriosus: A Comparison with Echocardiography. Neonatology 112 (3):231–237. 10.1159/000475774
  Reason: Insufficient hemodynamic data (data presented as mL kg^−1^ min^−1^)
10. Mutoh T, Sasaki K, Yamamoto S, Yasui N, Ishikawa T, Taki Y (2018) Performance of Electrical Velocimetry for Noninvasive Cardiac Output Measurements in Perioperative Patients After Subarachnoid Hemorrhage. Journal of neurosurgical anesthesiology. 10.1097/ana.0000000000000519
  Reason: Insufficient hemodynamic data (data presented as Cardiac Index)
11. Narula J, Chauhan S, Ramakrishnan S, Gupta SK (2017) Electrical Cardiometry: A Reliable Solution to Cardiac Output Estimation in Children With Structural Heart Disease. Journal of cardiothoracic and vascular anesthesia 31 (3):912–917. 10.1053/j.jvca.2016.12.009
  Reason: Insufficient hemodynamic data (data presented as Cardiac Index)
12. Schubert S, Schmitz T, Weiss M, Nagdyman N, Huebler M, Alexi-Meskishvili V, Berger F, Stiller B (2008) Continuous, non-invasive techniques to determine cardiac output in children after cardiac surgery: evaluation of transesophageal Doppler and electric velocimetry. Journal of clinical monitoring and computing 22 (4):299–307. 10.1007/s10877-008-9133-0
  Reason: Inconsistent data
13. 13. Song R, Rich W, Kim JH, Finer NN, Katheria AC (2014) The use of electrical cardiometry for continuous cardiac output monitoring in preterm neonates: a validation study. Am J Perinatol 31 (12):1105–1110. 10.1055/s-0034-1371707
  Reason: Insufficient hemodynamic data (data presented as mL kg^−1^ min^−1^)
14. Teefy P, Bagur R, Phillips C, Karimi-Shahri K, Teefy J, Sule R, Dempsey AA, Norozi K (2018) Impact of Obesity on Noninvasive Cardiac Hemodynamic Measurement by Electrical Cardiometry in Adults With Aortic Stenosis. Journal of cardiothoracic and vascular anesthesia 32 (6):2505–2511. 10.1053/j.jvca.2018.04.040
  Reason: Insufficient hemodynamic data (data presented as Cardiac Index)
15. Tirotta CF, Lagueruela RG, Madril D, Velis E, Ojito J, Monroe D, Aguero D, Irizarry M, McBride J, Hannan RL, Burke RP (2017) Non-invasive cardiac output monitor validation study in pediatric cardiac surgery patients. J Clin Anesth 38:129–132. 10.1016/j.jclinane.2017.02.001
  Reason: Insufficient hemodynamic data (data presented as Cardiac Index)
16. Trinkmann F, Berger M, Michels JD, Doesch C, Weiss C, Schoenberg SO, Akin I, Borggrefe M, Papavassiliu T, Saur J (2016) Influence of electrode positioning on accuracy and reproducibility of electrical velocimetry cardiac output measurements. Physiol Meas 37 (9):1422–1435. 10.1088/0967-3334/37/9/1422
  Reason: Duplicate publication of data
17. Trinkmann F, Schneider C, Michels JD, Stach K, Doesch C, Schoenberg SO, Borggrefe M, Saur J, Papavassiliu T (2016) Comparison of bioreactance non-invasive cardiac output measurements with cardiac magnetic resonance imaging. Anaesthesia and Intensive Care 44 (6):769–776
  Reason: Other tested method

## Appendix 7

See Fig. 7.

## Appendix 8

See Fig. 8.

## Appendix 9

See Fig. 9.
